# Invasive transmural fungal infection as a rare cause of thoracic aortic occlusion

**DOI:** 10.1016/j.jvscit.2025.102006

**Published:** 2025-10-08

**Authors:** Joanna F. Shaw, Benjamin Emert, Vincent Rowe, Jesus Ulloa

**Affiliations:** a Department of Surgery, Division of Vascular Surgery, UCLA School of Medicine, Los Angeles, CA; b Department of Pathology, UCLA School of Medicine, Los Angeles, CA

**Keywords:** Aortic mural thrombus, Mucormycosis, Angio-invasive infection, Aortic occlusion

## Abstract

Disseminated fungal infection resulting in aortic occlusion is a rare and highly morbid clinical phenomenon. This article reports the aortic occlusion of a 15-year-old male who had undergone recent emergent splenectomy and induction chemotherapy for newly diagnosed acute lymphoblastic leukemia. After initial stabilization, he developed progressive sepsis and ischemia, with cross-sectional imaging findings of pneumomediastinum and descending aorta intraluminal thrombus, which rapidly progressed to aortic occlusion. His clinical status continued to decompensate until he ultimately succumbed to his disease.

The patient was a 15-year-old previously healthy male who presented to his local hospital with spontaneous splenic rupture. He underwent emergency exploratory laparotomy with splenectomy, abdominal packing, and temporary closure, and was transferred to a quaternary hospital for higher level of care. His initial laboratory values from the local hospital were notable for a white blood cell count greater than 400 × 10^9^/L, a hemoglobin of 4.4 g/dL, and a platelet count of 37 × 10^9^/L. On arrival to the pediatric intensive care unit, the patient was intubated, sedated, and hemodynamically unstable on two vasopressor medications with an ongoing transfusion requirement. He was resuscitated before returning to the operating room with the pediatric surgery team for abdominal washout and primary closure.

On hospital day 2, the patient was diagnosed with B-cell acute lymphoblastic leukemia (B-ALL). By day 6, his clinical picture had improved; he was extubated to nasal cannula, no longer required vasopressor support, and at this time, induction chemotherapy was initiated. Several days later, the patient developed neutropenic fever of unknown infectious origin. He developed a large pleural effusion for which a chest tube was placed at the bedside, and a computed tomography angiography of ths chest was ordered to rule out venous thromboembolism in the setting of ongoing tachypnea and tachycardia. This computed tomography scan was significant for an aortic intraluminal irregularity, for which the vascular surgery team was consulted (Fig 1). The scan also revealed evidence of possible esophageal injury.

**Fig 1:**
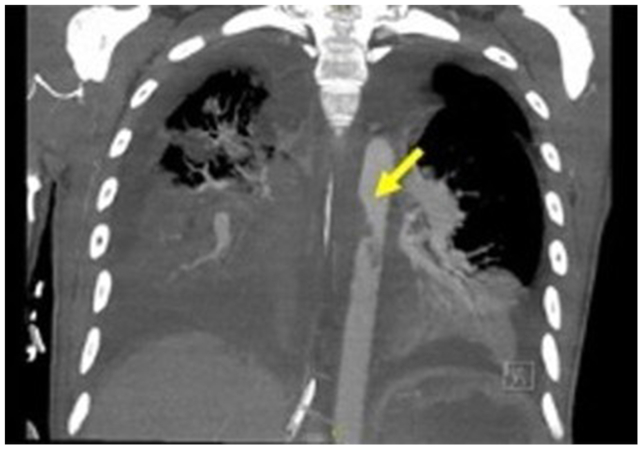
Initial computed tomography scan with aortic irregularity.

On physical exam, the patient was well-appearing with a right sided-chest tube in place, no abdominal pain, and with palpable pulses. Imaging review by the vascular surgery team showed no evidence of aortic dissection, pseudoaneurysm, or rupture. Initial management recommendations included therapeutic anticoagulation, emergent workup for esophageal injury, and repeat imaging 24 hours later to assess for evolution of aortic thrombus.

A repeat computed tomography scan with oral contrast confirmed esophageal perforation, for which the gastroenterology team performed an emergent esophagogastroduodenoscopy. The study was notable for ecchymotic tissue throughout the esophagus and stomach with a full thickness esophageal perforation at 25 cm. A covered esophageal stent was successfully placed. After the procedure, however, the patient remained intubated and required vasopressor support.

Three hours later, the patient significantly decompensated. He began having cardiac arrhythmias and ST elevations with elevated troponins, an increased oxygen requirement, and worsening lactic acidosis, coagulopathy, and hypothermia. A computed tomography angiography was repeated immediately and demonstrated occlusion of the descending thoracic aorta, mid-superior mesenteric artery, and new main pulmonary artery thrombus (Fig 2).

**Fig 2:**
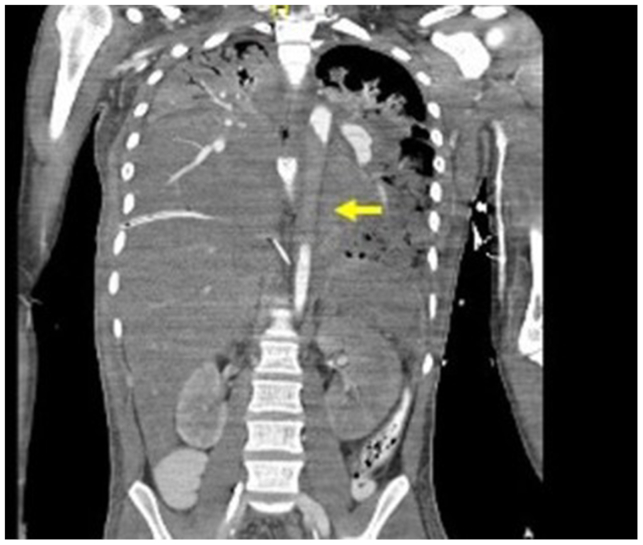
Repeat computed tomography scan demonstrating occlusion of the thoracic aorta.

The patient continued to physiologically decompensate, and a goals of care discussion was had with his family. Due to his inability to tolerate the physiologic demands of an invasive intervention, the decision was made to manage his condition without surgery. He was continued on a heparin drip, and developed bilateral lower extremity mottling to the level of the iliac crests, no pulses or Doppler signals on exam, and worsening neurologic status. His hospital course ended in mortality secondary to worsening multisystem organ failure.

An autopsy was completed, and revealed transmural fungal infection in the esophagus with extensive periesophageal fungal exudate, mural invasion of the thoracic aorta with an associated fungal thrombus (Fig 3), histological evidence of fungal hyphae, and no evidence of aortic dissection or perforation. Altogether, these findings suggest initial invasive fungal infection of the esophagus, leading to perforation and extension into the thoracic aorta, with subsequent dissemination into the pulmonary vasculature, right atrium, and inferior vena cava. The patient in this case report is deceased. His parents agreed with publication of case details and images.

**Fig 3:**
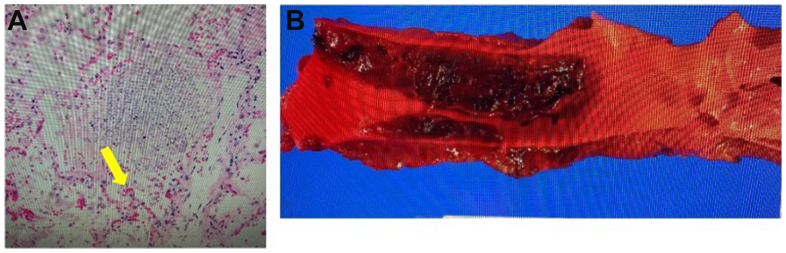
Histologic and gross pathologic specimens. **(A)** Histologic slide of broad, ribbon-like fungal hyphae of *Mucorales*. **(B)** Gross specimen of aortic thrombus.

## Discussion

Mucormycosis is an uncommon opportunistic fungal infection that primary affects those with diabetes mellitus or compromised immune systems, including those with hematologic malignancies or organ transplants.^1^ Information on disease prevalence is scarce due to its rarity and lack of a systematic reporting system, although one study estimated the cumulative incidence of mucormycosis at 16 per million person-years in the United States in 2016.^2^ Mucormycosis is a rapidly progressive disease with mortality as high as 70%, even when diagnosed and treated promptly.^3^ Antifungal medications and surgery are the only active agents against the pathogen and are recommended in treatment guidelines.^4^^,^^5^

Aortitis secondary to invasive disseminated fungal infection is an even rarer clinical entity. Although mucormycosis typically affects the respiratory system, the pathogen can spread contiguously to vessels and other structures in immunocompromised patients.^6^ The diagnosis of aortoinvasive mucormycosis is challenging due to its rarity, nonspecific symptoms which often include fever, chest or abdominal pain, and because its confirmation typically necessitates an invasive tissue biopsy.^7^ Several case reports of aortic involvement of mucormycosis infection have been reported in the literature, including one case of a 6-year-old female child with B-cell acute lymphoblastic leukemia, who developed aortitis with subsequent aortic rupture, and who survived after aggressive medical treatment and surgical debridement with complete replacement of the abdominal aorta and iliac arteries.^8^ In this case, the antifungal treatment for a presumed non-*Aspergilllus* invasive fungal infection was started before clinic deterioration and rupture of the aorta. Unfortunately, in the case of our patient, by the time his repeat imaging demonstrated significant aortic involvement, he was too critically ill to tolerate an invasive surgical intervention.

Other angioinvasive fungal infections such as aspergillosis have been reported in the literature as well, although the incidence appears quite rare; only 16 cases in immunocompetent individuals had been published by 2022.^9^ Typically, aspergillosis affects the lungs and causes pulmonary symptoms, whereas mucormycosis predominantly affects rhino-orbital-cerebral tissues.^10^^,^^11^ Aortic aspergillus infection is typically associated with aortic graft infection, with most cases thought to be due to airborne contamination of the graft before implantation.^12^ Like aortic mucormycosis, the diagnosis is often challenging with negative blood cultures in a critically ill patient, and tissue histopathology and culture remain the most sensitive mechanism for diagnosis.^13^ Optimal outcomes have been achieved with graft explanation, extra-anatomic bypass, and prolonged treatment with antifungal agents.^12^

## Conclusion

Angio-invasive fungal species are often difficult to diagnose and contribute to high rates of mortality among immunocompromised patients. Secondary procedures and interpretation of cross-sectional imaging may cloud clinical evaluation, which highlights the need for independent review of imaging by consulting vascular surgeons. Early consideration of fungal infection on the differential diagnosis for high-risk patients may facilitate medical care discussion, and help determine the role of operative interventions.

## Funding

None.

## Disclosures

None.
